# Supplementary material for: Experiences shaping research career intention among Black, Hispanic, and Indigenous-identifying first-year allopathic medical students in the United States: A qualitative study
Source: PLoS One. 2026 May 19;21(5):e0349227. doi: 10.1371/journal.pone.0349227 (PMC13186377; doi:10.1371/journal.pone.0349227)
Supplement: S1 Table — (DOCX) [file pone.0349227.s001.docx]

**S1 Table. Interview guide**

| **Domain** | **Question** | **Probes** |
| --- | --- | --- |
| **Baseline Professional Identity** | Can you tell me about when you first realized that you wanted to become a physician?  When you first realized you wanted to become a physician, what did you imagine that physicians do? What was their day-to-day job like?  How do you envision your own career as a physician? What roles will you have? | When? Who was involved?  How has this changed?  Are you interested in clinical work, teaching, research, administration?  Can you walk me through what a typical day would look like? |
| **Undergraduate Experiences** | Prior to medical school/during college, were there any specific experiences that impacted your thinking about your future career as a physician? Can you tell me about them? | Did you engage in research during undergrad?  [If yes] Can you walk me through your research experience?  How did research experiences as an undergrad shape your future career plans?  How did you find your research mentor?  Can you think of a particular experience with your mentor that impacted how you thought about your future as a physician? |
| **Application and Matriculation** | How did you decide where to matriculate for medical school? Can you walk me through your decision-making process? | Were there specific medical school characteristics or experiences that contributed to your decision? Can you tell me about them? |
| **First Year of Medical School**  Key domains: Interactions with faculty; interactions with students; interactions with senior leadership; research experiences; experiences of discrimination; mentorship; social network; competing life demands | How has the first year been?  Have there been any specific experiences that have influenced how you think about your future as a physician? New opportunities? Things that you definitely won’t do? Experiences confirming career intentions?  Do you have any mentors? Can you tell me about that?  Have you engaged in activities outside of the classroom that have influenced your thinking about your future career? (ie research, community service, leadership roles)? Can you tell me about that?  What has been your biggest challenge this year?  Can you recall a time when your race or ethnicity impacted your experience as a student?  Do you expect to be involved in research during your medical career? If yes, how much time do you expect to spend on research pursuits?  ***For students w/ no career interest in research & no mention of research in interview***  Has there ever been a time when you considered research as a component of your training or future career? Can you tell me about that?  Is there anything that you think could happen in your medical school training that would make you more likely to envision yourself as a physician-scientist in the future? | What coursework has influenced you?  Do you have a research mentor?  [if no mentor]: are you hoping to find a research mentor? Why or why not?  [if yes mentor]: why did you choose this mentor? Can you walk me through the process by which you found them? Can you tell me about the last time you met?  [if yes mentor]: have there been surprises/challenges  [if yes mentor]: have there been experiences with your mentor that have confirmed/challenged how you see your future career in medicine? If so, can you describe them?  Tell me about your experiences to date related to research during your time as a medical student.  [If involved in research] How did you get involved with research during your time as a medical student? What motivated your involvement in research?  [If not involved in research to date] What factors were relevant in not participating in research during medical school?  Before you started medical school last summer, what did you expect your research experience during medical school would look like?  What are you hoping to get from research experiences during medical school?  What type of support has helped you overcome challenges?  Have there been other challenges, e.g., financial concerns, lack of institutional support,  Do you feel like your medical school is a good fit for your educational needs so far? Why or why not? |
| **Closing** | Is there anything else you’d like to share with me about your experiences in your first year of medical school? |  |
