# Supplementary material for: Experiences shaping research career intention among Black, Hispanic, and Indigenous-identifying first-year allopathic medical students in the United States: A qualitative study
Source: PLoS One. 2026 May 19;21(5):e0349227. doi: 10.1371/journal.pone.0349227 (PMC13186377; doi:10.1371/journal.pone.0349227)
Supplement: S2 Table — (DOCX) [file pone.0349227.s002.docx]

**S2 Table. Code structure** (Version 8.16.24)

100: Awareness of Physician-Scientist Pathway

200: Research Career Intention

300: Factors Impacting Research Career Choice

400: Motivations for Research during Medical School

500: Research Training, Opportunities, and Experiences during Medical School

600: Mentorship During Medical School (Research or Non-Research)

700: Imposter Syndrome (experienced in medical school)

800: Sense of Belonging in Medical School

900: Diversity at Medical School (student and faculty) and Institutional/Peer Support of Students

1000: Intersectionality

1100: Experiences of Discrimination During Medical School

1200: Minority Tax

1300: Premed Experiences

1400: Medical School Selection and Application Process

1500: Great Quotes

| **Codes/Subcodes** | **Quotes** |
| --- | --- |
| **100: Awareness of physician-scientist pathway:**  Awareness of PS as a career option that involves skills and training as both a physician and scientific investigation | **100**: “My direct PI is someone who for a long time has you know, only worked a shift a week because the rest of his time is, is you know, tenure track research faculty as opposed to sort of the clinician educator or other, other tracks. So really all of my superiors were research concerned in some way, shape or form some early career, some mid-career, some very late career. So it was a really welcome sort of foyer into what the world of academic clinical medicine might look like and the various partnerships that exist sort of interdepartmentally, but then also with third parties. We worked on a few studies that were investigator-initiated collaborations with pharmaceutical companies or medical device companies as well as sort of through bread and butter academic clinical research. Mm-Hmm. So I thought *it was a really sort of nice broad introduction to sort of the scope of work that might be available to me if I go down that path.*”  **-ID 145** |
| **200: Research Career Intention (Yes/No):** A student's intention to pursue, or not to pursue, a physician-scientist pathway. (+ or -) | **200:** “but I hope that, like, again, my aspiration is for me to be very involved in research, specifically public health research” **-ID:18** |
| **300: Factors Impacting Research Career Choice:**  Any statement describing factors that influence a student’s decisions about a research career path | **300:** “I really like the research and have probably over committed myself already to some research work, took quite a bit of research work. So, you know, clinical, the, like, the avenues in clinical I'm sorry, in academic medicine are, you know, appealing and things that I'll certainly consider. *I think there's obviously a tremendous amount that I just don't know yet about sort of what those careers look like.* *But based on who I've worked with and the type of work that I'm interested in it's an, it's an area of interest”***- ID-145**  **300:** “I also noticed like there was very little black PIs or black people in research...” **-ID: 90**  **300:** “thinking more about my life outside of medicine, I'm not sure what I want to go into. But with this realization, I think I want a better balance between being like a professional and a woman outside of medicine”-**ID:90**  **300:** “I was really happy to learn of the variety of ways that one can craft and the level of flexibility in this profession that is pretty exciting.” **-ID:130** |
| **400: Motivations for research during medical school:** Factors that influence decision to pursue research while in medical school | **400**: “What I didn't know was how important research was in the field of medicine. And that was, that's something that I'm still like realizing to this day is a very important part of being or of like medical school in general, or like becoming a physician.”  - **ID: Pilot 1**  **400: “**I think that a big motivator to engage in research early on and at the level of dedication that I'm hoping to put into it is in part motivated by the knowledge that our residency track now has a huge reliance on research.”  **400**: “I really wanna try something new and like challenge myself in a way to summer. But also with this project in particular, I like the idea of it of applications in the future as far as fertility medicine goes. That really intrigues me. ” **-ID:1368**  **400: “**I don't know if that has to do more with finances as much as it has to do with kind of the type of work that I wanna engage in Yeah.” **ID- 130**  **400**. “Like, this is an easy paper if you just do it this way. But I guess I wasn't looking at like an easy paper or like an easy authorship. It was more like, we're not adding anything...there may be a push just to do like a lot of like meta reviews or something where you're not contributing. And I feel like it is the actual contribution that makes it worthwhile...” **-ID:1373** |
| **500: Research training, opportunities, and experiences during medical school:**  Availability of/access to research training and experiences engaging in research during medical school under a PI. | **500**: “I have also been admitted into this fellows cohort with a URiM serving medical kind of organization that I'm doing a community health project with. But all of those are things that I hope will ultimately yield some sort of publishable object or abstract or conference presentation.”  **500**: “A lot of begging and pleading via email (to find research opportunities). All the other ones I would just like constantly google like research opportunities or apply for like positions that I was unqualified for like a lab director and they would be like, okay, no. But I guess if you’re interested, you can be a lab tech”-**ID: 129**  **500**: **“**it is very different from undergrad. So it's been a much more, I guess on me a, a slower process of getting onboarded into that because you're, you have more concerns about, you know, how you're gonna study for your board exams you know, getting acquainted with everything else around. But now I am getting onto like, research studies and start slowly starting to like, help with data collection and, and whatnot.” **-ID 1373**  **500:”** I got involved in research in December on in one research project and I'm first author for that. And then I'm starting my summer research project and I'm first author for that too.”-**ID 2179** |
| **600: Mentorship during medical school (research or non-research):**  Guidance and support provided by faculty or peers, especially an experienced person. | **600:** “I sent it to that mentor and she told me like, reduce all the words that I had. She's like, people are busy, they don't wanna read all that. So that was really helpful.” -**ID:90**  **600:** “So one of them she is actually an African American female plastic surgeon in XXXX, and she's probably the only one in XXXX. So I definitely knew that as a minority woman that was trying to get into that field I needed to have access to someone who looked like me that was successful in that field. Why, say, she's not a research mentor is because she doesn't do research.” **-ID:Pilot 1**  **600: “**it's hard for me to really come by a mentor who also came from similar background.” -**ID:226**  **600:** “I always feel like upperclassmen who brings me on to their project are very willing to teach me they're willing to show me the ropes...I'm working on a research paper on, he was just showing, giving me tips and yeah, some tips and advice on writing a manuscript.” **-ID:226** |
| **700: Imposter syndrome (experienced in medical school):**  Persistent doubting of skills and abilities, despite evidence to the contrary. | **700:** “…when you go into these rooms, and you don't see anyone that looks like you, and you automatically feel like an imposter which I feel all the time, especially when I go to like conferences and stuff like that.” **-ID:Pilot 1**  **700:** “And I'm kind of trying to train myself to become a researcher. But because I also didn't have those skill sets. It's kind of putting that feeling of imposter syndrome in me. I don't necessarily think you need research to be a good physician like there's many fields in like specialties that don't even do research. And they're great physicians. But I guess, just for me personally like that was something that was a shock to me, because now feels like that dream is kind of far.” **-ID:Pilot 1** |
| **800: Sense of Belonging in medical school**  Feeling (or lack of feeling) of support, acceptance, inclusion, and identity as a member of a group. | **800**: “ … they sometimes act like this is for them and them only, and that you should like tread lightly and like only ask for certain things and like, not speak up when things are wrong. And if you do, it's almost kind of like pushed back on you. Like a club had put on a panel for people who had mashed into like women surgeons or whatever, and I'm part of the group and I said, I would love to see black women included next time.” -**ID:90** |
| **900: Diversity at the medical school (students and faculty) and institutional/peer support of students**  Characteristics of the institution, the role of or support from the institution or from its members (including students) in providing opportunities for success in medical school/career development. For example, support can be financial, academic, specific to URiM students or available to all students. | **900**: “a lot of my decision making was influenced by the experiences I had at those institutions. Sort of **** was very, very generous with financial aid. I was offered the, I think they just stopped giving the XXX scholarship, but the XXX is sort of an all-expenses paid situation.”  -**ID:1145**  **900**: “...trouble spots that I have with academic institutions about their diversity metrics. Yes. and one of the trouble spots that I have with it is that you will find probably close to a hundred percent of schools saying that diversity is important to them. But it shapes out in a couple of different ways depending on, on, on where you're at in the country and that kind of stuff. I feel very fortunate that I think I attend a very diverse institution. Like my, my class certainly feels like a reflection of the country as well as a reflection of the dire need that there are in shortages of racial and ethnic minorities...But I know that's not the case in many different places.”**-ID:130**  **900**: “...students are having a better time or like a happier time compared to some other schools... I feel like there's a lot of opportunities to do a bunch of things.” **ID:130**  **900**: “So there is a this is like a student as well as faculty and staff led committee. It's called the **steering Committee. It's for identity and inclusion. Pretty much it is like an extension of DEI I think I would say. And it's really about how we can come up with programming that will help celebrate the different i, the intersectional identities we have here at X.” -**ID:226**  **900**. Yeah. I love talking to other students. And this sounds kind of vain, but like getting like validation. So like I'll talk to like another student and like, I, like, I feel really behind. I feel like I and not cut up at all. And they're like, me too. Like, I'm really behind too. Like and so just like being able to kind of like take a, a bigger, like like to zoom out a little bit and see that like I'm not the only one that's like behind or feeling kind of like stressed about the pace helps every single time. Every single time.-**ID18** |
| **1000: Intersectionality**  Any mention of student identities in addition to race and ethnicity, including (but not limited to) immigration status, gender & sexual orientation, that may lead to discrimination or disadvantage. Including **SES.** | **1000:** “I grew up in Nigeria, so I was exposed to a lot of health disparities when I grew up. So I knew I wanted to do something in the health field.” **- ID: Pilot 1**  **1000:** “But I didn't know that when I was applying to college because my parents didn't go to college...”-**ID:90** |
| **1100: Experiences of discrimination during medical school:**  Experience of being treated unfavorably due to age, disability, ethnic origin, race, gender, religion/belief, or sexual orientation**.** | **1100:** “I could feel the difference in like how he would respond in a way. And in terms of entitlement, like just some white kids in the class, I mean, I don't know if all of them are white, but white presenting <laugh> students, it's just like, they sometimes act like this is for them and them only, and that you should like tread lightly and like only ask for certain things and like, not speak up when things are wrong. And if you do, it's almost kinda like pushed back on you.” **-ID 90**  **1100:** “like constantly proving to my colleagues that, okay, this guy is, is actually competent and he knows what he's talking about.” **-ID 1722**  **1100:** “And I was like, didn't I just say that? Like right off the bat, I knew what you were talking about, but because I said it, you didn't think that was right. And then it's funny 'cause that person went to Tufts undergrad and I had gone out to dinner with one of our mutual friends who knew that person. And my, our mutual friend was saying like, yeah, like he said that you're so smart and blah, blah, blah. And I'm like, yeah, but then you also don't listen when I tell you what I think.” **-ID 90** |
| **1200: Minority Tax (not necessarily research-related**): URiM students spend more time and effort on certain activities compared with non-URiM students, often needing to sacrifice academic excellence in order to fulfill these additional diversity duties. | **1200**: “...we were kind of shoeboxed into that because now when we go to the safety net hospitals, it's just assumed that her and I are gonna take on the Spanish speaking patients...just because we speak Spanish, that they're using us to, to translate and yeah. Using us to talk to those patients.”  **ID-1950** |
| **1300: Premed experiences**  Research or clinical experiences prior to medical school,  or any premedical experiences directly related to medical school/career in medicine, e.g., advice received from advisors about pursuing a career in medicine/getting into medical school, exposure to URiM physician role models. Do not code medical school selection/application (see 1400). | **1300**: “My role was mostly like that of like, I would say like a medical assistant in a way. Or we were supposed to find a way to help the office either by like starting a new project there or just something to help the office too while we were there. But I gotta see a lot of like what nurses got to do and like behind the scenes saying like checking in patients, doing vitals, getting vaccines. I loved getting to do that and like work at the nurses and also work with the doctors.” **-ID:1368**  **1300**: “And then there was one school I remember it was like, okay, just like, tell me important key points about yourself so I can note it down like a bullet, like I'm a bullet, you know, bullet point checklist for them to like present to their committee whether or not to accept me. And things like that definitely deterred me away from those schools because it, it just felt like they didn't even put an effort to really get to know me. And XXX was one of those schools where through interview, through the interview process, it feels like they wanted me as much as I wanted them. And at last, when the financial aid package finally came out XXX offered me full tuition.”**-ID: 226**  **1300**: “So going into college I had a mentor who kind of opened the doors in a way for me to be involved with research through the research program that she had spearheaded at ** And it's specifically trying to motivate like underrepresented students in health, just health in general to pursue healthcare in a grad at a graduate level where like on a professional level.”**-ID: 226**  **1300**: “my advisor kind of like talks about a TED talk that he listened to and he was like, you know, sometimes people confuse what they're passionate about versus what they should actually like do in life... I was just like, you know, I'm absolutely not gonna tell this guy anything else that I wanted to do” - **ID: 1722** |
| **1400: Medical School Selection and Application Process**: Any mention of the thought process involved with and the actions taken toward applying to medical schools and accepting a spot in a specific schools class | **1400: “**For me, it really came down to money. What school is gonna offer me the, the, the money I needed? 'cause I, like I told you before, I, I got accepted to several schools, but it was like, if none of them offered money, then I couldn't go in the first place. Right. So really when people ask me what was the top choices, whatever school was willing to offer financial aid or even, or even hinted that they would give some sort of support while you're there, you know, because just getting there is, is one thing, but then being able to support you through your full force that you actually become a successful and be candidate is also important. That's also important for residency.” -**ID:1373** |
| **1500: Great Quotes:**  Statements that reflect deep insights; great quote to include in manuscript | **1500**: “After a while you just get accustomed to just being in a shell and not saying anything.” - **ID: 1722** |
