# Supplementary material for: Experiences shaping research career intention among Black, Hispanic, and Indigenous-identifying first-year allopathic medical students in the United States: A qualitative study
Source: PLoS One. 2026 May 19;21(5):e0349227. doi: 10.1371/journal.pone.0349227 (PMC13186377; doi:10.1371/journal.pone.0349227)
Supplement: S3 Table — (DOCX) [file pone.0349227.s003.docx]

**S3 Table. Additional quotes supporting each theme and subtheme**

| **Theme/Subtheme** | **Additional Example Quotes** |
| --- | --- |
| *Theme 1:* Structured premedical research exposure was described as pivotal to developing early research engagement and interest in research careers.  *Subtheme*: Dedicated research offices or programs that support underrepresented students and/or those unfamiliar with research facilitated engagement in experiences students regarded as essential for becoming acclimated with research.  *Subtheme:* While students with premedical research exposure reported early interest, barriers such as not being valued as collaborators, lack of acknowledgement, or limited attention from premedical research mentors dampened interest in pursuing research. | So when I got into undergrad there was this research group that reached out... they were for underrepresented students... they just wanted to see how interested were you in research? Are you willing to do this for the next four years of your life? And I was like, Why not? You know what I mean? I've never had formal training, if anything like that. So when I got in, they basically gave you like a list of labs that you might... be interested in... my first year [of undergrad] I got introductory lectures for neuroscience. And I was like, you know, I'm pretty interested in that, so let's see how that is. I connected with the PI, and they just kind of invited me on board... And I was like, okay, this is something I can probably stay in... like, okay, this is pretty cool. This is not something that I ever grew up seeing or anything. So I really liked that experience. (ID 1373: Black male)  I did like a... research program my last year as well... that informed [that] I don't just have to be like a physician. I can be a physician scientist... and being confident in that as well... that program really helped me... we wrote a whole paper, we presented our research, like an oral presentation at an undergrad research symposium. (ID 292: Hispanic female)  The first PI I ever had, yeah, he wasn't [always] a mentor... I felt like I was more like a lab technician versus actually doing research, learning the science, and what I'm doing. I did learn a lot of valuable skills there, but I feel like, in terms of understanding and practicing the research aspect, I don't feel that I got a lot of mentorship in that sort of realm, I would say. (ID 1891: Hispanic female) |
| *Theme 2:* Research orientations reflected a commitment to using research as a vehicle for social justice and community impact | I was kind of just interested in anything like health disparity related [when deciding on research project options in medical school]… if there's any research I do want to do in the future…It'll probably be some form of public health research or clinical trial research… (ID 837: Asian/NHPI male)  I'm pretty interested in research, teaching and, mentorship…Doing translational basic science research…you have to get people that have no idea about your project to understand from a bird's eye view what you're working on. So having that skill, I think is really helpful when you think about working inside a low socio-economic community where your patients might not really have a great educational background… (ID 1722: Black male)  [When student was asked how they envision their future career as a physician]: And then also research because I do believe it's severely lacking in the underserved, especially in the Black community. (ID 1664: Black female)  I was thinking very specifically, like equity research. I do see myself, no matter where I am, kind of like wanting to be involved with that, just because I think it's something that can be done well, or it can be done, you know, poorly, or just be done for the sake of being done. And because of the experience [participant detailed an instance of discrimination just before this statement] that I have too. That's something I would wanna continue being involved with. (ID 1604: Black/Hispanic female) |
| *Theme 3:*  High -quality research mentorship was characterized by authentic relational investment, skill development, and the distinct value of racial and ethnic identity-concordant role models.  *Subtheme*: Mentor-mentee racial and ethnic identity concordance fostered belonging, encouragement, and a trusted space to share experiences, expanding students’ vision of themselves as researchers. | She took, you know, two hours out of her day to sit down and show me how to read a research paper, like truly read it. And I know that she taught me well… it was just her patience. She cared about me as an individual... So she just really put in the effort, I think, to become a good mentor. (ID 1365: Hispanic female)  “She's been helping me along the way, whether it be academically or…it’s been emotionally a lot of times where I'm like, I can't do this...She's just always there to reassure me and just affirm that this is for me...” (ID 18: Black male)  “I got along really well with the attending that [participant’s medical school] paired me up with…He's actually calling me after this on his way home from work to give me career advice…He's amazing. I know his wife and kids now. And so that's been such an amazing opportunity for me to have like somebody in the career that I want to go into that's just taken me under his wing.” (ID 404: AIAN/White female)  “There's a scholarship as far as research goes this summer and she's helped me like build my resume for that…I have no experience bench work, so this is really new to me, but like she's been very patient and understanding in that and wants me to really learn.” (ID 1368: Black/Hispanic female)  Representation matters because I feel like having mentor who kind of understands where you're coming from and your lived experience on the firsthand, on the firsthand really does make a difference. And I also feel more encouragement from them…I hope that they understand that they had to do a lot more to get to where they are compared to like a student who's overrepresented in medicine. (ID 226: Asian/NHPI male)  I always try to envision how my life is gonna look like as a doctor. And so it's a little hard to do that when I'm envisioning it through the lens of someone that does not look like me. [having a Black male mentor] it's shown me and demonstrated to me a lot of the barriers that I thought were barriers aren't actually barriers. And they show me a lot of like that I can be in a lot of spaces. And I can do a lot of things, like research, which is one of them. And so it's, it's helped so much in just being able to just, just show me what I'm capable of. (ID 18: Black male)  “There wasn't a single Black advisor, there wasn't a Black teacher, there wasn't a research coordinator...there was no one doing research…not saying that the people that were there were not supportive because they were…but it's still like feeling that ‘other’ feeling.” (ID 1664: Black female) |
| *Theme 4:* The research arms race for residency placement was described as amplifying systemic inequities that constrained students’ research engagement.  *Subtheme:*  Research productivity and prestige metrics for residency conflicted with students’ values and amplified structural inequities such as the minority tax and financial constraints.  *Subtheme:* Inequities in social capital constrained students’ navigation of research pathways and contributed to feelings of marginality within medicine. | “Research is kind of like a have-to, if you wanna do a competitive specialty…I went to a meeting two months ago where they set the average number of publications that someone in plastic surgery has to 27. Wow. And so that kind of like was super stressful…It's like, okay, do you have time to study, to get a on everything [referring to leadership positions in student groups], and also be a part of these research projects? Like you can't let one thing slack…Right now it [research] is definitely not one of like the top things that I'm like interested in pursuing.” (ID 129: Black female)  “Because I know I wanna go into surgery and because I'm considering orthopedics, which is a very like a pretty competitive field to get into. I kind have to do a lot of extra like resume builders just to show more initiative. So, research is taking a lot of time. I got involved in research in December in one research project and I'm first author for that. And then I'm starting my summer research project and I'm first author for that too…And then leadership wise…I'm stretching myself too much…” (ID 2179: Hispanic female)  And so there are a lot of projects that I'm like, I, this is over my head, or like, I'm only working on the introduction, or I'm only helping with the manuscript writing. I had no part in study design or implementing it. And so I'm not; I don't have a personal attachment to it. But I wanna work on it…to get ahead or to just have another publication on my CV. (ID 1021: Black female)  “We're so pressured to like get research, to be in organizations, to be involved and be active and volunteer, but also learning…My biggest challenge is taking the step back…” (ID 1979: Hispanic female)  “People from underrepresented communities when they enter spaces or institutions where it's kind of an anomaly or a privilege for them to be there, they find themselves to be extra burdened because they need to do all of the responsibilities expected of them as a student, but then also other things that will bolster their applications for residency, but then are also expected to do this extra work of giving back to the community that got them there…I've personally felt it…” (ID 837: Asian/NHPI male)  “Social capital—just like being able to like reach out to a lot of people, like if your parent was a physician, has connections at the school that you're at and introduces you…maybe you could reach out to those people and ask them questions or inquire about research and things like that. And you have more of an idea of things that you need to do to get to their position. Whereas I think somebody like my case, you really have to go outta your way to try to make those connections. And I think that that's tough in itself…when you're trying to do everything that everybody else is doing, but also make up for your lack of social capital…” (ID 1722: Black male) |
