# Supplementary material for: Experiences shaping research career intention among Black, Hispanic, and Indigenous-identifying first-year allopathic medical students in the United States: A qualitative study
Source: PLoS One. 2026 May 19;21(5):e0349227. doi: 10.1371/journal.pone.0349227 (PMC13186377; doi:10.1371/journal.pone.0349227)
Supplement: Attachment — Submitted filename: Reviewer Report.docx [file pone.0349227.s005.docx]

**Reviewer Report**

This manuscript examines the early experiences that shape research career intentions among first-year medical students who are underrepresented in medicine, using a qualitative approach. The topic is timely, relevant, and well aligned with current priorities in medical education and workforce diversification.

**Strengths:**
The study design is appropriate for the research question, and the qualitative methodology is clearly described and rigorously applied. Sampling, data collection, and analysis are well justified, and the use of rich participant quotations strengthens credibility. The analytic process demonstrates reflexivity, use of an audit trail, and assessment of information power, all of which enhance trustworthiness. The findings are coherent, well-organized, and meaningfully situated within the existing literature. Conclusions are appropriately drawn and not overstated. The manuscript is clearly written, logically structured, and presented in standard English.

**Areas for Minor Revision:**
The primary issue relates to data availability. While supplementary materials and the coding framework are provided, full access to de-identified participant data is restricted and contingent upon request and completion of the parent longitudinal study. This does not fully align with PLOS ONE’s data-sharing policy. The authors are encouraged to either (a) provide a clearer justification for these restrictions in accordance with journal policy or (b) consider mechanisms to improve transparency and accessibility while maintaining participant confidentiality.

**Recommendation:**
Minor Revision. The manuscript represents a strong contribution to the literature, and addressing the data availability clarification would further strengthen its suitability for publication.
