# Supplementary material for: Experiences shaping research career intention among Black, Hispanic, and Indigenous-identifying first-year allopathic medical students in the United States: A qualitative study
Source: PLoS One. 2026 May 19;21(5):e0349227. doi: 10.1371/journal.pone.0349227 (PMC13186377; doi:10.1371/journal.pone.0349227)
Supplement: Attachment — Submitted filename: Response to Reviewers.docx [file pone.0349227.s007.docx]

|  | **Department of Emergency Medicine** |
| --- | --- |
|  | Translational Research Building |
|  | 227 East 30th Street, 1st floor, NY, 10016 |
|  | T 475-241-5322  E [shruthi.venkataraman@nyulangone.org](mailto:shruthi.venkataraman@nyulangone.org) |
|  |  |

April 10, 2026

Saima Aleem, PhD
Academic Editor
PLOS ONE

Dear Dr. Aleem,

Thank you for you review of our manuscript, “Experiences shaping research career intention among Black, Hispanic, and Indigenous first-year medical students in the United States: a qualitative study.” We appreciate your and the reviewers’ thoughtful comments and recommendations and have responded to each concern and suggestion below:

**JOURNAL REQUIREMENTS**

1.Please ensure that your manuscript meets PLOS ONE's style requirements, including those for file naming. The PLOS ONE style templates can be found at <https://journals.plos.org/plosone/s/file?id=wjVg/PLOSOne_formatting_sample_main_body.pdf> and

<https://journals.plos.org/plosone/s/file?id=ba62/PLOSOne_formatting_sample_title_authors_affiliations.pdf>

**Response:** We have edited the manuscript to ensure that it meets the PLOS ONE style requirements, including for file naming.

2. Please include a complete copy of PLOS’ questionnaire on inclusivity in global research in your revised manuscript. Our policy for research in this area aims to improve transparency in the reporting of research performed outside of researchers’ own country or community. The policy applies to researchers who have travelled to a different country to conduct research, research with Indigenous populations or their lands, and research on cultural artefacts. The questionnaire can also be requested at the journal’s discretion for any other submissions, even if these conditions are not met. Please find more information on the policy and a link to download a blank copy of the questionnaire here: https://journals.plos.org/plosone/s/best-practices-in-research-reporting. Please upload a completed version of your questionnaire as Supporting Information when you resubmit your manuscript.

**Response:** We have now included a completed version of the PLOS ONE questionnaire on inclusivity in global research as Supporting Information and referenced it in the manuscript text, as requested.

***Modified text (lines 212-214):*** *“Additional information regarding the ethical, cultural, and scientific considerations specific to inclusivity in global research is included in the Supporting Information (S3 Checklist).”*

3. Thank you for stating the following financial disclosure: “This study was funded by the National Institute of Health (NIH), project number R35GM153263. The NIH did not have any role in the study outside of funding. The authors listed are all independent from the NIH and had full access to all the data in the study and can take full responsibility for the integrity of the data and the accuracy of the data analysis.” Please state what role the funders took in the study. If the funders had no role, please state: "The funders had no role in study design, data collection and analysis, decision to publish, or preparation of the manuscript." If this statement is not correct you must amend it as needed. Please include this amended Role of Funder statement in your cover letter; we will change the online submission form on your behalf.

**Response:** The funders had no role in the study. We have now included the suggested statement verbatim in our cover letter.

4. In the online submission form you indicate that your data is not available for proprietary reasons and have provided a contact point for accessing this data. Please note that your current contact point is a co-author on this manuscript. According to our Data Policy, the contact point must not be an author on the manuscript and must be an institutional contact, ideally not an individual. Please revise your data statement to a non-author institutional point of contact, such as a data access or ethics committee, and send this to us via return email. Please also include contact information for the third-party organization, and please include the full citation of where the data can be found.

**Response:** We have amended our data statement to comply with both the PLOS ONE Data Policy and the terms of the verbal informed consent obtained from study participants.

***Modified text (‘Data Availability’ statement):*** *“Due to the highly sensitive nature of the data collected in this study, the data cannot be made publicly available in order to protect participant confidentiality and to comply with the terms of the verbal informed consent obtained from participants. At the time of consent, participants were informed that access to their information would be restricted to the research team and individuals responsible for research oversight, and that the data would not be shared with any parties outside the investigative team, including program leadership at their respective institutions. Questions regarding data access or participant protections may be directed to the Yale University Human Research Protection Program (HRPP@yale.edu).”*

5. Please include your full ethics statement in the ‘Methods’ section of your manuscript file. In your statement, please include the full name of the IRB or ethics committee who approved or waived your study, as well as whether or not you obtained informed written or verbal consent. If consent was waived for your study, please include this information in your statement as well.

**Response:** We have included a full ethics statement with the full name of the IRB who approved our study and explained our informed verbal consent process in the ‘Methods’ section, as requested.

***Associated text (lines 137-144): “****Recruitment ended on May 9, 2024. Interested participants were sent the consent form (detailing the study aims, voluntary nature of participation, confidentiality protocols etc.) for review in advance of their interview. During the interview, the interviewer read and reviewed the consent language with the participant, asked if there were questions or answered questions and asked if they agreed to proceed with the interview. Participants were informed that they could choose not to participate (with no professional or other consequences) and choose to end the interview at any time. We documented that date informed verbal consent was obtained in REDCap. The Yale University Institutional Review Board approved all study procedures.”*

6. Please include captions for your Supporting Information files at the end of your manuscript, and update any in-text citations to match accordingly. Please see our Supporting Information guidelines for more information: <http://journals.plos.org/plosone/s/supporting-information>.

**Response:** We have now included captions for the Supporting Information files at the end of the manuscript (see lines 841-844) and updated in-text citations (see lines 150, 180, 213-214, 216, 217) to match accordingly.

7. If the reviewer comments include a recommendation to cite specific previously published works, please review and evaluate these publications to determine whether they are relevant and should be cited. There is no requirement to cite these works unless the editor has indicated otherwise.

**Response:** The reviewers and editor did not make such recommendations.

8. Please review your reference list to ensure that it is complete and correct. If you have cited papers that have been retracted, please include the rationale for doing so in the manuscript text, or remove these references and replace them with relevant current references. Any changes to the reference list should be mentioned in the rebuttal letter that accompanies your revised manuscript. If you need to cite a retracted article, indicate the article’s retracted status in the References list and also include a citation and full reference for the retraction notice.

**Response:** We have reviewed the reference list for completeness and accuracy. To our knowledge, we have not cited any retracted articles. However, we found a duplicated reference (# 14 and # 45 in the previous version) and have remediated the duplication in the revision.

**REVIEWER’S COMMENTS**

**Reviewer #1:**

1. One area that would benefit from further attention is data availability. While supplementary materials are provided, access to the full de-identified dataset is restricted and contingent upon request and completion of the parent longitudinal study. Greater alignment with PLOS ONE’s data-sharing policy, or a clearer justification for the stated restrictions, would strengthen transparency.

**Response:** We appreciate this important point. We have now amended the Data Sharing statement to reflect greater adherence with PLOS ONE’s data sharing policy and the terms of the verbal informed consent obtained from study participants.

***Modified text (‘Data Availability’ statement):*** *“Due to the highly sensitive nature of the data collected in this study, the data cannot be made publicly available in order to protect participant confidentiality and to comply with the terms of the verbal informed consent obtained from participants. At the time of consent, participants were informed that access to their information would be restricted to the research team and individuals responsible for research oversight, and that the data would not be shared with any parties outside the investigative team, including program leadership at their respective institutions. Questions regarding data access or participant protections may be directed to the Yale University Human Research Protection Program (HRPP@yale.edu).”*

**Reviewer #2:**

1. First, the manuscript draws on several relevant concepts, including social cognitive career theory, identity concordance, minority tax, social capital, and values alignment. While all are appropriate, the Discussion occasionally assigns these concepts a stronger explanatory role than warranted by a qualitative descriptive design (mainly through words such as drives, determines, shapes etc.). I would recommend clarifying that these frameworks function as interpretive lenses rather than analytic or causal models, and slightly nuancing language that implies determination or directionality.

**Response:** We thank the reviewer for this insightful and constructive feedback. As suggested, we have nuanced and softened the causal-sounding language in the ‘Discussion’ as well as throughout the manuscript. We have also added a line in the ‘Limitations’ to explicitly clarify the epistemic limits of the work and findings.

***Modified text (lines 522-524):*** *“Our findings highlight four interconnected domains of experience that participants described as salient in how they made sense of early research engagement and research career possibilities…”*

***Modified text (lines 546-549):*** *“Participants described structured research opportunities as especially important in supporting their confidence and navigation of research spaces, particularly for students who perceived themselves as lacking prior exposure or professional networks.”*

***Modified text (lines 571-574):*** *“Identity-concordant mentorship was described by participants as particularly meaningful in fostering a sense of belonging and possibility within research spaces, aligning with literature showing that representation supports persistence in STEM and academic careers.[28,36]”*

***Modified text (lines 604; paragraph title):*** *“A sense of value misalignment and research disengagement”*

***Modified text (lines 605-608):*** *“While our findings span distinct experiential domains, a consistent thread was participants’ references to perceived value incongruence when reflecting on their early research experiences, which influenced how they articulated enthusiasm, ambivalence, or hesitation towards future research engagement.”*

***Modified text (lines 631-634):*** *“Fourth, consistent with a qualitative descriptive approach, the concepts referenced such as social cognitive career theory, identity concordance, minority tax, social capital, and values alignment, are used to interpret participants’ accounts rather than to imply causal relationships or predict research career trajectories.”*

***Modified text (lines 674-676):*** *“This qualitative study offers an in-depth examination of experiences first-year URiM medical students described as informing their engagement with research and how they weighed the possibility of physician scientist careers.”*

***Modified text (lines 678-679):*** *“…and structural barriers intensified by residency-driven publication pressures influenced how they engaged with research and evaluated research careers.”*

2. Second, the study's contribution and novelty could be made more explicit, for example, by briefly and clearly stating what this study adds beyond existing work, particularly the focus on the first year of medical school which is rarely examined, the early emergence of value misalignment with academic research culture, and the way residency competitiveness is already shaping research engagement at this stage of training.

**Response:** We appreciate this thoughtful comment and have added statements in the ‘Discussion’ and ‘Conclusion’ to explicitly highlight the more novel findings of this study.

***Modified text (lines 529-538):*** *“These findings extend the literature by capturing how these experiences unfold at a formative period seldom examined in depth, particularly via qualitative enquiry. While prior work has examined URiM-specific barriers to academic medicine careers more broadly,[11,24] few studies have focused on the early medical school period or investigated how RCI specifically begins to emerge and evolve…Notably, even at this early stage of their training, participants reflected on their research experiences using interpretive lenses of values alignment, structural inequity, and residency competitiveness.”*

***Modified text (lines 585-587):*** *“Our findings suggest that pressures associated with these benchmarks (which our study participants were acutely aware of) are already influencing how students approach and evaluate research as early as the first year of medical school.”*

***Modified text (lines 614-616):*** *“Notably, students described these value tensions as emerging during the first year of medical school, highlighting that questions about alignment with academic research culture can arise early in medical training.”*

***Modified text (lines 679-682):*** *“Notably, even at this early stage of medical training, students described tensions between their personal values and prevailing academic research culture, framing research less as a meaningful pursuit and more as a strategic obligation.”*

3. Finally, the participant and public involvement section is a strength, but could be slightly streamlined to maintain a descriptive rather than evaluative tone. At the moment, it sounds more like advocacy, whereas the normative statements in this section would fit better in the Discussion.

**Response:** We thank the reviewer for this helpful observation. In response, we revised the Participant and Public Involvement section to maintain a more neutral, descriptive tone focused on clearly documenting how URiM students were involved across stages of the study (e.g., refinement of research questions, recruitment, data collection, analysis, and manuscript preparation). We removed or avoided evaluative and normative language and limited this section to factual reporting of participant involvement. We believe these revisions better align the section with conventions for reporting participant and public involvement.

***Modified text (lines 202-210):*** *“URiM medical students were involved at multiple stages of this study. The project was conceived by academic medicine faculty engaged in mentoring URiM medical students and informed by their observations of student engagement in research. URiM students contributed to the refinement of research questions and served as the primary study participants. URiM students also participated in recruitment, conducted interviews, contributed to coding and analysis, and collaborated in authoring and revising the manuscript. The interview guide was piloted with a small group of URiM students to assess clarity and burden. All participants were financially compensated and invited to share feedback at the conclusion of their interviews, though they did not provide feedback on the study findings.”*

Thank you for considering our revised manuscript. We appreciate the reviewers’ thoughtful feedback and would be happy to provide any further clarification if needed. We look forward to your decision.

Sincerely,

Shruthi Venkataraman, MD, MS

Postdoctoral Research Fellow, Boatright Lab

NYU Grossman School of Medicine
